# The study of biosafety risk identification and analysis for facilities in biosafety level 3 laboratories

**DOI:** 10.3389/fcimb.2025.1658759

**Published:** 2025-08-27

**Authors:** Bingwang Lv, Zhiyu Zhou, Jinsong Wang, Qun Niu, Yuchen Shi, Yanbo Sun, Hongda Zhao, Dongsheng Lu, Tao Fan, Yang Huang

**Affiliations:** National Institutes for Food and Drug Control, Beijing, China

**Keywords:** biosafety level 3 laboratory, facility, biosafety, risk identification, risk analysis

## Abstract

**Objective:**

The aim of this study was to identify and analyze biosafety risk points in biosafety level 3 (BSL-3) laboratory facilities to bring awareness to the attention of administrative staff, reduce the biosafety risks, and improve the risk management.

**Methods:**

The biosafety risk points in BSL-3 facilities were identified by literature searches and field research methods, and the identified biosafety risk points subsequently analyzed using the fault analysis event tree method.

**Results & conclusion:**

Throughout the comprehensive screening and identification of biosafety risk points in BSL-3 laboratory facilities, risk assessments were performed to rank their seriousness. This will help effectively reduce the biosafety risk level of BSL-3 laboratory facilities.

## 1 Introduction

The first standard BSL-3 laboratory in China was built in 1987. At present, there are only a few dozen BSL-3 laboratories recognized by the China National Accreditation Service for Conformity Assessment. Costs associated with BSL-3 laboratory facilities under construction and in operation are currently high, as key facilities and equipment are imported from abroad. Because BSL-3 work in China started relatively late, biosafety awareness and knowledge in operating a BSL-3 facilities are lacking and coupled with few available specialists. Because of this, ensuring the safety and stability of such facilities for long-term laboratory operations is a huge challenge for facility managers and an urgent problem needing to be solved (Mourya et al., 2014; Dickmann et al., 2015).

Stringent BSL-3 laboratory practices and procedures are crucial for preventing biosafety-related accidents and ensuring the safe operation of the laboratory. In addition to other controls, such as elimination, administrative controls, and use of personal protective equipment (PPE), normally and stably operating facilities and equipment significantly contribute to keeping biological safety risks within an acceptable range (Odetokun et al., 2017). China generally focuses on biosafety risks caused by laboratory personnel, experimental activities, and equipment, with less emphasis on the management and maintenance of the laboratory facilities. However, according to statistics on the causes of laboratory controlled biological agent escape events in the United States from 2003 to 2009 by the U.S. Centers for Disease Control and Prevention (CDC) in 2010, mechanical failures of facilities and equipment and protection failures accounted for 5.82% of all causes (National Research Council, 2011).

The research on biosafety risk mainly includes three aspects: biosafety risk identification, biosafety risk analysis, and biosafety risk management and control. The main difficulty in biosafety risk management and control is identifying and analyzing risks, which is the basis and focus of all risk management procedures. Therefore, to improve the safety and stability of BSL-3 laboratory operations, the aim of this article is to research the biosafety risk identification and analysis methods associated with BSL-3 facilities. This article helps to provide a more comprehensive, in-depth understanding of the biosafety risk points of BSL-3 facility systems and operation.

## 2 Materials and methods

### 2.1 Biosafety risk point identification of the uninterruptible power supply system

The UPS system is one of the most important facility systems in BSL-3 laboratories. According to the provisions of the People’s Republic of China national standard 50346–2011 Technical Code for the Construction of Biosafety Laboratories, the BSL-3 laboratory and the ABSL-3 laboratory should be powered according to the Class I load. A level I load power supply refers to having two normal power sources that supply power. When one normal power source cannot supply power, then the other normal power source can switch automatically to supply power. Additionally, the national standard also stipulates that the UPS system shall be adopted for particularly important loads, and the power supply time shall not be less than half an hour. For example, the ABSL-3 laboratory of Class B2 must be powered according to the first level load (Achitectual And Technical Code For Biosafety Laboratories, 2004). Lastly, the standard provides that extremely important loads not only require the UPS system, but also the electric generator, and the UPS should supply power normally until the startup of the electric generator (Achitectual And Technical Code For Biosafety Laboratories, 2004). If the UPS system has a fault during the process of an experiment and the municipal electric power is cut off, then the experimenter may potentially exposed. Therefore, the UPS system’s function has significant meaning in biosafety protection for BSL-3 laboratories and is an important biosafety risk control point.

The risk failure point of the UPS system is mainly associated with the safety and stability of the direct current and alternating current capacitors in the battery and UPS cabinet, respectively. The optimal temperature of the battery in the UPS system is 20–25 °C. If the battery temperature is too low, its capacity will be reduced. If the temperature is too high, the battery life will be reduced (Wentao, 2020). The batteries commonly used in the UPS system are a valve regulated, lead-acid batteries. The service life is generally divided into two types: 5 years and 10 years. The recommended service life of a 5-year battery is 3 to 4 years, with a professional inspection suggested every 6 months. The recommended service life of a 10-year battery is 6 to 8 years, with a professional inspection also recommended every 6 months (Shibao, 2022). The key components of the UPS cabinet are alternating current (AC) capacitors, electrolytic capacitors, and fans. The expected service life is more than 7 years, with recommendations including replacements every 5–6 years and annual inspections. In addition, the regular safety inspections should include dust removal and charging/discharging operations in the UPS machine room. Proper maintenance of the UPS system can ensure its smooth and normal operation system (Shufang, 2022).

The sealing of the pipes that carry the conduit for the power supply system is also a biosafety risk point that should be focused on. If infectious materials are exposed and the pipes are not properly sealed, then an infectious substance divulging event may occur. The power distribution pipeline of the BSL-3 laboratory shall be laid with metal pipes. When the power distribution pipeline passes through the wall or floor, it shall meet the airtightness requirements of the laboratory. Airtight parts, sleeves, and special cables through devices shall be installed through the wall. The casing should also be airtight, and non-shrink and non-flammable materials should be used for sealing (Zaki, 2010).

Overall, the biosafety risk points for the BSL-3 laboratory UPS system include the tightness of the power supply pipeline, the airtightness of installations through the wall, ensuring important equipment is connected to the UPS system, and the stability and reliability of the UPS system itself. Among these, the tightness of the power supply pipeline and airtight effects of relevant parts need to be tested and examined in the annual BSL-3 laboratory verification testing procedure, which usually uses the smoke testing method. If the laboratory is not refurbished, the annual laboratory test can maintain the airtightness up to the standard for one year (Achitectual And Technical Code For Biosafety Laboratories, 2004). The operational reliability of the UPS system is an important biosafety risk point, and is critical for ensuring the safety of laboratory personnel and samples. If the laboratory suddenly loses power and the UPS simultaneously fails, it will lead to serious biosafety consequences.

### 2.2 Biosafety risk point identification of heating ventilating and air conditioning system

**Figure 1:**
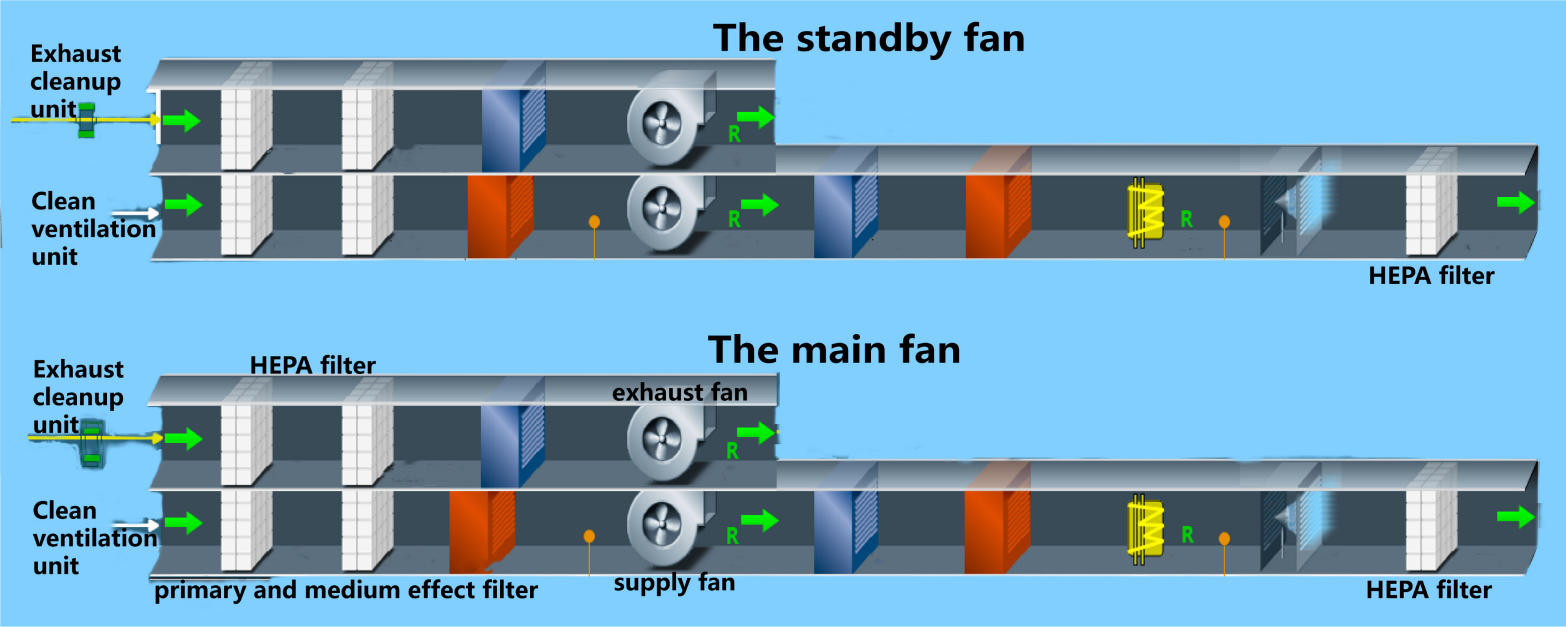
The diagrammatic drawing of the heating ventilating and air conditioning system.

The heating ventilating and air conditioning system in a BSL-3 laboratory refers to the fresh air system that filters outdoor air through primary, intermediate, sub advanced, and advanced air, with settings for temperature, humidity, pressure, and other conditions to quickly purify and filter potentially polluted air. The heating ventilating and air conditioning system systems are generally composed of an air supply air conditioning unit, air exhaust conditioning unit, air filtration system, and air cooling and heating humidification system (Yong, 2009). This system is the most important for ensuring the safety of the experimental environment by providing clean air with no pollution, maintaining lab negative pressure, and preventing potential leakage of infectious or toxic substances from the BSL-3 laboratory (Wurtz et al., 2016). The diagrammatic drawing is shown in the **Figure 1** below.

The heating ventilating and air conditioning system is critical for preventing highly pathogenic microorganisms from leaking and spreading outside the BSL-3 laboratory. Its working principle is negative air pressure isolation (Yan, 2019), which prevents dangerous agents from spreading to the core area and spaces outside the prevention and control area by using the ventilation and air conditioning unit system to establish a negative pressure gradient and directional airflow (Zhuang and Jinsong, 2005; Zongxing et al., 2010). The heating ventilating and air conditioning system is the largest, most complex, and most important system among the facilities and systems in the BSL-3 laboratory. Because this system is involved with many facilities and equipment, it also affects many control indicators, such as the ventilation rate, air volume, pressure difference, high efficiency filtration, and air temperature and humidity. When identifying biosafety risk points, each piece of equipment should be examined for every link from the fresh air entry point to where air is discharged. After efficient filtration, the air outlet should remain in the downwind direction of the dominant wind (relative to the air supply outlet) and should be at least 12 meters away from the air supply outlet, 2 meters higher than the top of the building, and have a corresponding biosafety protection design (Laboratories-General requirements for biosafety, 2008).

The biosafety risk points of the air conditioning purification system mainly include the performance of the high-efficiency air filter, the airtightness and corrosion resistance of the closed valve at the interface of the air supply and exhaust ducts, the switching condition of the standby fan, the stability of the air volume and differential pressure regulation system, the airtightness of the animal isolation equipment, and the connection with the exhaust system. During the annual inspection and audit of the BSL-3 laboratory, the high efficiency filter requires an *in situ* leak detection test. The air supply and exhaust pipe interfaces and airtightness of the isolation equipment and exhaust system also need to be verified (Nolte et al., 2021): Importantly, the daily use and maintenance process, stability of the air volume and differential pressure regulating system, and working condition switching of the standby fan should be debugged and maintained regularly to ensure the stability and safety of the system (World Health Organization 2005).

### 2.3 Biosafety risk identification of the building maintenance structure

The building maintenance barrier structure, as the secondary barrier of the BSL-3 laboratory, is the basic structure meant to ensure that infectious materials in the laboratory are not leaked. Many BSL-3 laboratories in China are constructed inside existing buildings, and most of these buildings are of level II structural safety. (Level II structural buildings are considered “general buildings”, and if they are damaged, then the consequences can be serious). For newly built BSL-3 laboratories, the safety level should be level I. (Level I structural buildings are considered “important buildings”, and if they are damaged, then the consequences can be very serious.) As far as possible, the seismic fortification category should be based on the special fortification category, and the foundation design should be level A.

All gaps in the enclosure structure of BSL-3 laboratories should be free of visible crevice. When the relative negative pressure of the room is maintained at -250 Pa, the amount of air leaked in the room per hour should not exceed 10% of the net volume of the room under test (ABSL-3). The wall and ceiling materials of BSL-3 laboratories should be corrosion resistant and crack free, all windows should be closed windows, and the glass should be impact resistant and shatter resistant (Laboratories-General requirements for biosafety, 2008). The Class B2 main laboratory and its buffer room in ABSL-3 laboratories should adopt airtight doors. There are many key points for the construction of barrier environmental facilities, as well as many biosafety risk points (Jiuxiang, 2021). These key points include clean decoration, HVAC, water supply and drainage, gas power, electrical engineering, low voltage system (generic cabling, intercom, monitoring, access control), and automatic control engineering. These facilities can be installed in the wall through airtight parts to ensure the airtightness of the laboratory.

### 2.4 Identification of biosafety risk points in the central control system

The central control system of the BSL-3 laboratory includes many subsystems that mainly include the air conditioning automatic control system, communication system, alarm system, and video monitoring system. The central control system is the most complex system among all these in the BSL-3 laboratory, and generally consists of two upper graphic workstations, two master and slave servers, ethernet switches, WINCC (Windows Control Centre) configuration software, FDA certification software, industrial PLC (Programmable Logic Controller) controllers and I/O (Input/Output) interface modules, various sensors, electric valves, frequency converters, and alarm devices (Yong, 2009). The central control system controls vast majority of the BSL-3 laboratory, and its reliability plays a key role in the determine the overall biosafety of the laboratory.

The biosafety risk points of the central control system are primarily associated with the danger-hidden points(a place where may occur accident) of the sensors, valves, frequency converters, alarm devices, and other equipment it controls. Therefore, the central control system focuses on the stable operation and normal communication of the machine of the upper workstation, server, and ethernet switch to ensure that the sensors of the field equipment can transmit the field temperature, humidity, and room pressure parameters to the PLC controller in a timely and normal manner. The actuator is responsible for receiving the PLC controller signal to control the field equipment, and the PLC controller and I/O interface module receive the signal from the field sensor. After calculation, the control signal is output to the actuator to control the cleanliness, temperature, and humidity, room differential pressure, alarm display, and other functions of the air conditioning unit. Through the communication between the WINCC configuration software of the upper workstation, the data information stored in the server, and the PLC controller through the Ethernet switch, the control unit of the PLC is on the network, the upper workstation is the master station, and all the lower PLCs are slave stations. The communication between the workstation and PLC completes the monitoring of the entire system.

### 2.5 Identification of biosafety risk points in the water supply, drainage, and gas supply systems

The water supply, drainage, and gas supply system of the BSL-3 laboratory is an indispensable and critical facility system to facilitate experimental activities. Because the water supply, drainage, and gas supply systems need to connect and flow inside and outside the protection area, its safety and sealing play a decisive role in ensuring the normal progress of experimental activities. When infectious materials were exposed and the pipes were not properly sealed, then an infectious substance divulging event may occur from the gas/water pipes.

The biosafety risk points of the water supply and drainage system mainly include the airtightness, non-leakage, temperature resistance, pressure resistance, and corrosion resistance of the pipeline, the cutoff water tank should be set in ABSL-3, and the forced shower device should be set in the protection area. The water supply pipeline in the protection area should be set with maintenance valves and check valves for the main laboratory, and the water seal depth of the trap and floor drains should be set in the protection area according to the pressure difference requirements. When the sanitary ware without trap in the structure is connected to the drain pipe, the trap must be set below the drain outlet, the water seal of the drain pipe must be filled with water or disinfectant, and the main laboratory should be equipped with independent drainage branch pipes and valves. Importantly, the vent pipe of the drainage system in the protection area should be set separately and equipped with a high efficiency filter or other reliable disinfection device, with adequate ventilation.

## 3 Results and analysis

### 3.1 Biosafety risk analysis of the UPS system

**Figure 2:**
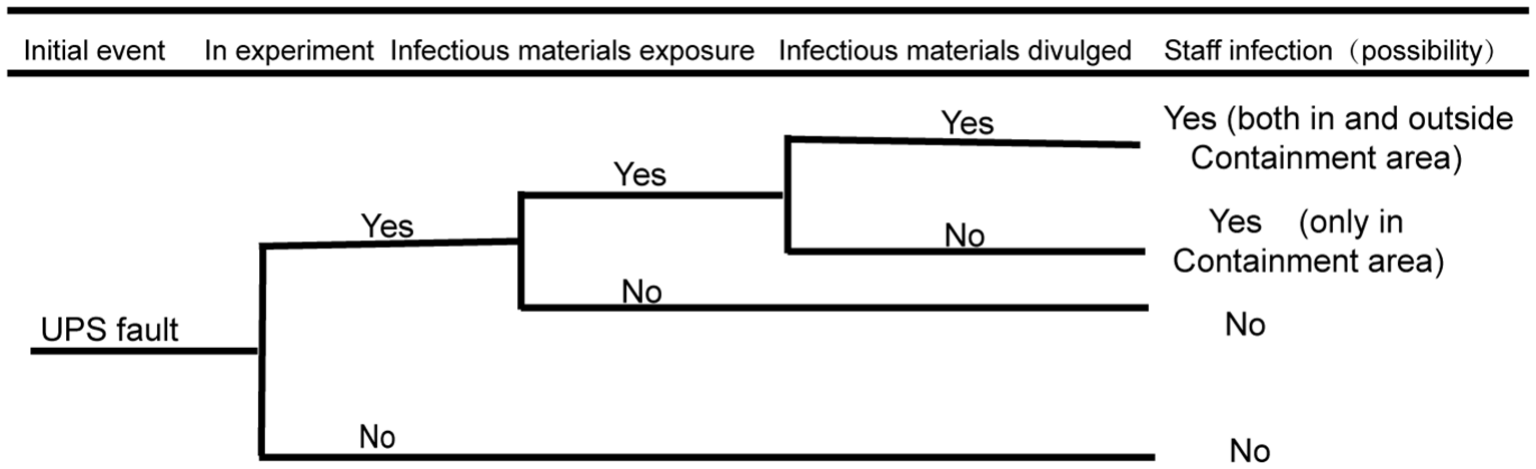
Fault analysis event tree of UPS.

As stipulated in the national standard 50346–2011 Technical Code for Building Biosafety Laboratories and in the actual scientific research work, the stability of the UPS system has an important impact on biosafety risks. As shown in **Figure 2** below, the biosafety risk of the UPS system is analyzed by using the event tree analysis method: the initial event refers to the important risk points in the development process when the biosafety accident does not occur. When both main power supplies are cut off and the UPS system fails to supply power; if a researcher is conducting an experimental operation with infectious materials in the biosafety cabinet, the researcher in the protection zone may be at risk of exposure; if infectious materials leak from protective barriers, such as biosafety cabinets, there is a biosafety risk of infection for personnel inside the protection area. If a problem or error occurs with the UPS system, there are potential risks for the personnel both inside and outside the containment area to be exposed. Additionally, this may contaminate the surrounding environment and possibly infect residents within the nearby community. The event tree analysis is shown in the figure below.

### 3.2 Biosafety risk analysis of the heating ventilating and air conditioning system

**Figure 3:**
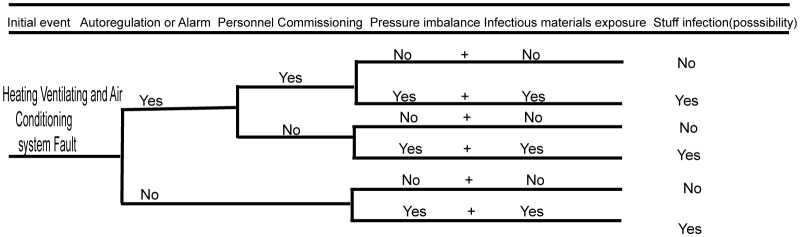
Fault analysis event tree of heating ventilating and air conditioning system.

The heating ventilating and air conditioning system plays an important role in BSL-3 laboratories, which also suggests that serious biosafety accidents may occur if the system fails (Wen et al., 2014). The biosafety risk of the heating ventilating and air conditioning system is analyzed using the event tree analysis method below (as shown in **Figure 3**). When the ventilation and air conditioning systems fail and the automatic control system does not automatically adjust or sound an alarm, then infectious materials can potentially leak. If, after automatic adjustment or alarm, maintenance management personnel do not perform appropriate mitigating actions, then the agent could be released if it was being wrongly manipulated or if a spill occurred outside of containment. If the ventilation and air conditioning systems have big faults, then there may be huge risks of infecting the residents in the nearby community and polluting the environment. The specific event occurrence analysis is shown in the figure below.

### 3.3 Biosafety risk analysis of the building maintenance structure

**Figure 4:**
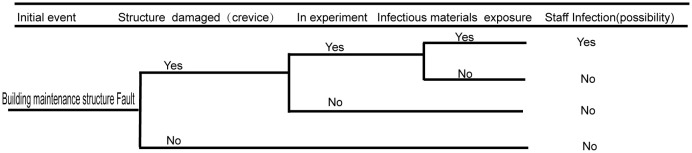
Fault analysis event tree of building maintenance structure.

Damage to the building maintenance structure is likely to impact the laboratory sealing. If the sealing of the building maintenance structure is compromised, leaks can potentially occur. A broken building maintenance structure can affect the personnel working or living in the community, as well as possibly pollute the environment. The detailed fault analysis event tree is shown in **Figure 4**.

### 3.4 Biosafety risk analysis of the central control system

**Figure 5:**
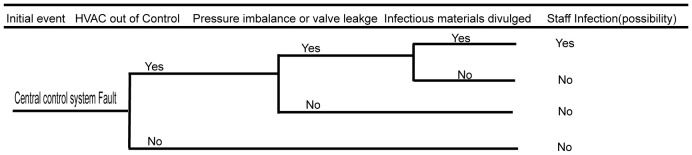
Fault analysis event tree of central control system.

The central control system is the most complex system in the BSL-3 laboratory. Once a failure occurs, it is likely to cause serious biosafety risks. In the central control system, the control of the purification air conditioning system has the greatest impact on the biosafety risk. Failure of the air conditioning system, loss of negative pressure in the protection area, or control failure of the key biosafety airlock valve may lead to serious biosafety consequences. The faults of the central control system can only affect the personnel working in the BSL-3 facility, which lowers the risk of polluting the environment and nearby community. The detailed fault analysis event tree is shown in **Figure 5**.

### 3.5 Biosafety risk analysis of the water supply, drainage, and gas supply systems

**Figure 6:**
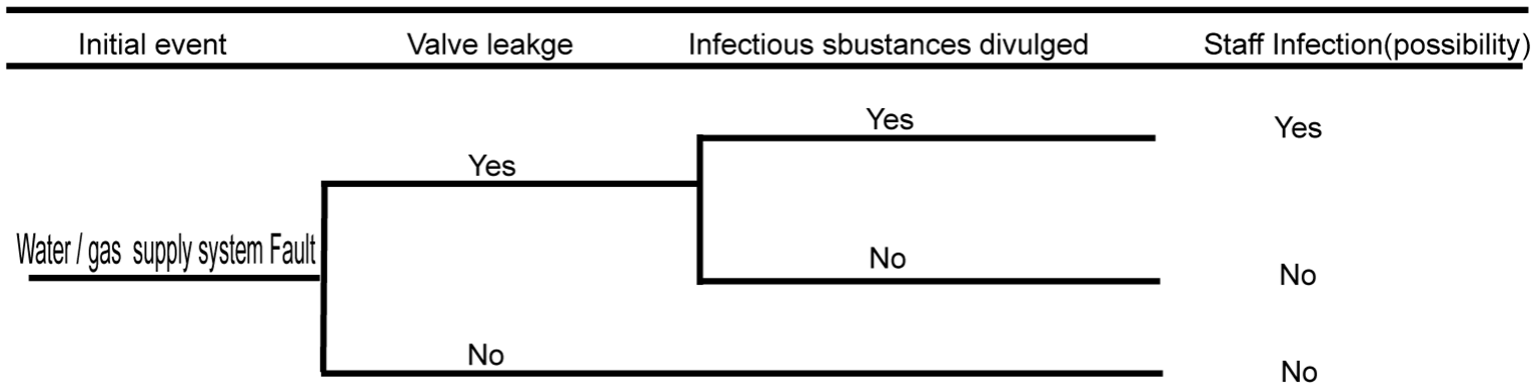
Fault analysis event tree of water/gas supply system.

The water supply, drainage, and gas supply systems run through the connected protection area and play key roles in ensuring the normal operation of the laboratory and experimental activities. Therefore, when gas or liquid in the pipeline of the protection area leaks or reverses, there will be a biological security risk of infectious substance leakage. Faults in the water/gas supply system have enormous potential risks to pollute the municipal tap water and air near the community. Hence, there is a huge risk of infecting people within the community. The detailed fault analysis event tree is shown in **Figure 6** below.

## 4 Discussion

The identification and analysis of these biosafety risk points are of great help to risk management. Therefore, after the identification and analysis of risk points in each system, we need to carry out the next step of risk management. For the uninterruptible power supply system, we need to do two power failure tests every year to ensure that the UPS system can normally start and run for at least 30 minutes in the state of power failure. In addition, it is necessary to carry out comprehensive testing of components such as batteries, capacitors, heat dissipation fan, air filter, control cabinet and circuit every year, and inspect the battery and machine room every day. For the heating ventilating and air conditioning system, in addition to routine maintenance inspections, it is essential to conduct periodic fault simulation testing. When we simulate the failure of the main fan, the standby fan can automatically operate and the whole system can run normally. For the building maintenance structure, we should conduct gap inspection and tightness test every year to ensure the integrity of the building maintenance structure. For the central control system, in addition to routing inspection, we should also regularly conduct a comprehensive inspection of the upper computer, software, PLC controller, sensor, actuator and other components to ensure the normal operation and control of the central control system. For water supply, drainage, and gas supply systems, check valves and High Efficiency Particulate Air filters need to be checked regularly to prevent failure.

In addition, these facility systems: the UPS system, heat ventilation and air conditioning system, building maintenance structure, central control system, water supply, drainage, and gas supply systems, interact with each other directly or indirectly. If the municipal power is off, the UPS system could support the central control system and heat ventilation and air conditioning system. Furthermore, the central control system can alert and warn the administrator if other systems stop functioning correctly. The building maintenance structure supports all of these systems. Although the primary purpose of this paper is to study the biosafety risks of facilities, facility management hinges on human factors—particularly for BSL-3, which require cross-disciplinary expertise to serve in distinct management capacities. Only through seamless, shoulder-to-shoulder collaboration among personnel who manage these facilities can the smooth and safety operation of BSL-3 laboratories be ensured.

## 5 Conclusion

Through a comprehensive analysis of biosafety risk point identification in BSL-3 laboratory facilities, we found that these risks are ubiquitous and can have a significant impact. In this article, we identified the biosafety risk points of these facilities. This study provides a more in-depth understanding of the biosafety aspects of these laboratories and summarizes the biosafety hidden dangers of each facility. We also included a biosafety risk analysis of the facilities and the possible consequences of the biosafety risk points using the fault tree analysis method. The failure of the risk points of some facilities can seriously affect the safe operation of the laboratory. Therefore, it is very important to conduct comprehensive, in-depth analyses of biosafety risk points for facilities. This research plays an important role in promoting the level of biosafety management, improving the understanding of biosafety risks, and supporting the safety management of BSL-3 laboratories. Through this study, we hope managers, laboratory personnel, and maintenance personnel will pay closer attention to the safe operation of BSL-3 laboratory facilities, improve the understanding of the specific biosafety risk points, and improve their management of these facilities.

## Data Availability

The raw data supporting the conclusions of this article will be made available by the authors, without undue reservation.

## References

[B1] Achitectual And Technical Code For Biosafety Laboratories (2004). (China construction heating & Refrigeration) 5, 6.

[B2] (2008). Laboratories-General requirements for biosafety. (Beijing, China: National Standardization Administration).

[B3] Dickmann P. Sheeley H. Lightfoot N. (2015). Biosafety and biosecurity: A relative risk-based framework for safer, more secure, and sustainable laboratory capacity building. Front. Public Health 3. doi: 10.3389/fpubh.2015.00241, PMID: 26539427 PMC4612646

[B4] Jiuxiang Y. (2021). The key points design and construction of experiment facility for laboratory animal about barrier environment. Contamination Control Air-Conditioning Technol. 4), 92. doi: 10.3969/j.issn.1005-3298.2021.04.023

[B5] Mourya D. Yadav P. Majumdar T. (2014). Establishment of Biosafety Level-3 (BSL-3) laboratory: Important criteria to consider while designing, constructing, commissioning & operating the facility in Indian setting. Indian J. Med. Res. (New Delhi India: 1994) 139, 171., PMID: 25297350 PMC4216491

[B6] National Research Council (2011). Protecting the frontline in biodefense research:the special immunizations program. Available online at: http://www.nap.edu/catalog.php?record_id=13112. 24983071

[B7] Nolte K. B. Muller T. B. Denmark A. M. (2021). Design and construction of a biosafety level 3 autopsy laboratory. Arch. Pathol. Lab. Med. (1976) 145, 407. doi: 10.5858/arpa.2020-0644-SA, PMID: 33307551

[B8] Odetokun I. A. Jagun-Jubril A. T. Onoja B. A. (2017). Status of laboratory biosafety and biosecurity in veterinary research facilities in Nigeria [. .Safety Health at Work 8, 49. doi: 10.1016/j.shaw.2016.08.002, PMID: 28344841 PMC5355539

[B9] Shibao Y (2022). Common malfunctions and countermeasures of hospital UPS batteries. Chin. Hosp. Architecture Equip. 23, 79. doi: 10.3969/j.issn.1671-9174.2022.02.013

[B10] Shufang S. (2022). Analysis on the management and maintenance countermeasures of UPS power supply in radio&TV machine room. Integrated circuit Appl. 39, 248. doi: 10.19339/j.issn.1674-2583.2022.03.111

[B11] Wen Z. Yang W. Li N. (2014). Assessment of the risk of infectious aerosols leaking to the environment from BSL-3 laboratory HEPA air filtration systems using model bacterial aerosols. Particuology 13, 82. doi: 10.1016/j.partic.2012.11.009, PMID: 38620193 PMC7148691

[B12] Wentao M. (2020). Discussion of the selection principle and maintenance method of UPS battery. Technol. Innovation Appl. 36), 101. doi: 10.19981/j.cn23-1581/g3.2020.36.041

[B13] World Health Organization. (2005). Laboratory biosafety manual. (World Health Organization) (06), 473.

[B14] Wurtz N. Papa A. Hukic M. (2016). Survey of laboratory-acquired infections around the world in biosafety level 3 and 4 laboratories. Eur. J. Clin. Microbiol. 35, 1247. doi: 10.1007/s10096-016-2657-1, PMID: 27234593 PMC7088173

[B15] Yan Z. (2019). Analysis of the simultaneous debugging case of air conditioning system in biosafety level 3 laboratory. Clean air conditioning Technol. 03), 91. doi: 10.3969/j.issn.1005-3298.2019.03.020

[B16] Yong S. (2009). Research and application of air conditioning purification control system in biosafety laboratory (Xian, Shanxi Province, China: Xidian University). doi: 10.7666/d.y1668746

[B17] Zaki A. N. (2010). Biosafety and biosecurity measures: management of biosafety level 3 facilities. Int. J. Antimicrob. Ag 36, S70. doi: 10.1016/j.ijantimicag.2010.06.026, PMID: 20801002

[B18] Zhuang W. Jinsong L (2005). Potential environmental influence of biosafety level three laboratory and its protective countermeasures. Proc. Acad. Military Med. Sci. 29, 263. doi: 10.3969/j.issn.1674-9960.2005.03.018

[B19] Zongxing Z. Yanju L Jiancheng Q (2010). Negative pressure difference control in airtight high-level bio-safety laboratory. China Saf. Sci. J. 20, 116. doi: 10.3969/j.issn.1003-3033.2010.06.020

